# The Isolation and Molecular Characterization of an Astrovirus From “Yellow” Chickens, China

**DOI:** 10.3389/fvets.2020.581862

**Published:** 2020-10-27

**Authors:** Wei Zhao, Zongyi Wu, Yongxiu Yao, Aijian Qin, Kun Qian

**Affiliations:** ^1^Ministry of Education Key Lab for Avian Preventive Medicine, Yangzhou University, Yangzhou, China; ^2^Jiangsu Key Lab of Preventive Veterinary Medicine, Yangzhou University, Yangzhou, China; ^3^The International Joint Laboratory for Cooperation in Agriculture and Agricultural Product Safety, Ministry of Education, Yangzhou University, Yangzhou, China; ^4^The Pirbright Institute & United Kingdom-China Centre of Excellence for Research on Avian Diseases, Pirbright, United Kingdom; ^5^Institute of Comparative Medicine, Yangzhou University, Yangzhou, China

**Keywords:** astrovirus, chicken, isolation, sequence analysis, molecular characterization

## Abstract

Chicken astrovirus (CAstV) infection is strongly associated with kidney disease, gout, “white chicks” hatchery disease, and runting and stunting syndrome (RSS). In the present study, 82.5% of 154 clinical samples from different provinces in China were positive for CAstV by RT-PCR. One CAstV isolate, designated as AAstV/Chicken/CHN/2017/NJ01, was successfully isolated from the small intestine of “Yellow” chickens using LMH cells. The genome sequence and structure analyses revealed that NJ1701 had the typical characteristics of avian astroviruses which was genetically distinct from other *Avastrovirus*. This isolate was classified as Group B subgroup i based on phylogenetic analysis of complete ORF2 (capsid) amino acid sequences. Meanwhile, growth depression and hatchability reduction were observed in the chicken embryo infection experiment. The results in the current study will contribute to our understanding of chicken astrovirus in China.

## Introduction

Astroviruses, belonging to the *Astroviridae*, are non-enveloped with a positive-sense, single-stranded RNA genome. The family *Astroviridae* is divided into genus *Mamastrovirus* including viruses that infect mammals and genus *Avastrovirus* including viruses that infect avian species (1–3). The virus particles are 28–30 nm with a star-like morphology. Although human astrovirus was first described in 1975, the morphology of the virus particles was not described until 2008 (4, 5). According to all published full-length sequences, the general genome organization of astroviruses is arranged in the order of 5′-non-coding region, ORF1a/ORF1b, ORF2, and 3′-non-coding region. The astrovirus non-structural proteins are encoded by ORF1a and ORF1b together, including a serine protease, a viral genome-linked protein (VPg), and an RNA-dependent RNA polymerase (RdRp). ORF2 encodes the capsid protein (CP), and the genetic distances of amino acid based on the analysis of the aa sequence of ORF2 is usually used for astrovirus species demarcation (1, 6).

Based on the different hosts infected by the virus, the *Astroviridae* family is divided into two genera: *Mamastrovirus* and *Avastrovirus* (1, 7). The astroviruses from the genus *Avastrovirus* are isolated from birds including chickens, turkeys, ducks, geese, and wild birds (8, 9). Five different astrovirus divisions have been identified in avian species: turkey astrovirus type 1 (TAstV-1), turkey astrovirus type 2 (TAstV-2), avian nephritis virus (ANV), chicken astrovirus (CAstV), and duck astrovirus (DAstV) (8). Recently, the International Committee on Taxonomy of Viruses officially divided the *Avastrovirus* genus into three species, namely, *Avastrovirus 1* (turkey), *Avastrovirus 2* (chicken), and *Avastrovirus 3* (duck) (9). Increasing evidence reveals that CAstV have strong associations with diseases of young birds such as runting and stunting syndrome (RSS), kidney disease with visceral gout, and “white chicks” hatchery disease in different countries and regions of the world (1, 2, 9–11). Some of the CAstV strains can transmit either horizontally or vertically (9). In addition, the diversity of CAstV and cross-species transmission between turkeys and mink have been reported (12). However, except for two reports on serological investigation and complete genome sequence analysis (13, 14), little is known about the prevalence of CAstV in China. Here we report the first isolation of a chicken astrovirus from a “Yellow” chicken flock in China using LHM cells, the molecular characterization of the virus by complete genome sequencing, and infection experiment in chicken embryos with the isolate.

## Materials and Methods

### Tissues and Cells

One hundred fifty-four fresh small intestines of 1-day-old chicks were collected from 10 different chicken flocks in Guangdong, Jiangsu, Anhui, and Shandong Provinces during 2017 to 2019. All of the selected flocks have mild growth problems. The LMH (chicken hepatocellular carcinoma epithelial cell line, CRL-2117, ATCC) cells were grown in DMEM/F-12 (Dulbecco's Modified Eagle Medium/Nutrient Mixture F-12) supplemented with 10% fetal bovine serum (FBS), 100 U/ml of penicillin, and 100 g/ml of streptomycin at 38.5°C in a 5% CO_2_ atmosphere.

### Viral Nucleic Acids Detection

Total RNA and DNA were extracted from tissues and cells using Trizol reagent (Invitrogen, Shanghai, China) and QIAmp DNA Mini Kit (Qiagen, Shanghai, China), respectively, according to the manufacturer's instructions. The RT-PCR for CAstV was performed as previously described (15). The 510-base pair (bp) fragment from CAstVs was amplified using the degenerate primers listed in Table 1 and sequenced by Gene Script Company (Nanjing, China). In order to rule out the presence of other common enteric viruses in chickens, the CAstV (NJ1701) was evaluated by PCRs and RT-PCRs specific for avian nephritis virus (ANV), reovirus, rotavirus, fowl adenovirus Group I (FAdV-1), infectious bronchitis virus (IBV), chicken parvovirus (ChPV), and Newcastle disease virus (NDV) as previously reported (16).

**Table 1:** Primer pairs used in this study.

**Primer**	**Product**	**Sequence**	**Product size**	**Source**
CAstV-510F	ORF1b partial	KCATGGCTYCACCGYAADCA	510 bp	Smyth et al. (15)
CAstV-510R		CGGTCCATCCCTCTACCAGATTT		
CAstV 1F	ORF1a partial	GAGGGTGTGGGCGATGGC	947 bp	This study
CAstV 947R		GCTGTTCACTATTAAAAGCACTACG		
CAstV 890F	ORF1a partial	AAGTGCTACAACACTCATGGGAACG	2,176 bp	
CAstV 3066R		TCCATTCCGCGTGATGGTCTCAA		
CAstV 3038F	ORF1b partial	GAGGTTAAATTTGAGACCATCACGC	1,196 bp	
CAstV 4123R		CCCTCGTTTCTGTCATATTTTTCAT		
CAstV 4118F	ORF1b partial	CCATGTTCGATCAGGACCAGAATT	1,187 bp	
CAstV 5625R		TGTAACTGCCATGCGATCATGTATT		
CAstV 4999F	ORF2 complete	CGGGATCCATGGCCGATAAGGCTGGGCCG	2,214 bp	
CAstV 7212R		CGGAATTCCTACTCGGCGTGGCCGCG		
CAstV 7169F	Non-structural	CTGAGCAGCAAAAACAACCT	326 bp	
CAstV 7495R		AAATGCCAATTAATTTAATTCAAAA		

### Virus Isolation

The homogenate of tissues from a “Yellow” chicken flock with mild growth problem was filtered through a 0.22 μm filter. The filtrate (0.5 ml) was inoculated onto LMH cultures with 80% confluency in six-well plates. After 3 h post incubation, the supernatant was replaced with normal LMH cell culture medium. After 3 days incubation, the cells underwent three freeze-thaw cycles, and the supernatant was directly inoculated onto new LMH cultures. The viral gene and antigen expression were detected by quantitative reverse transcriptase polymerase chain reaction (qRT-PCR) and immunofluorescent assay, respectively. CAstV isolate NJ1701 obtained by the fifth passage in LMH cells was used for further studies. After centrifugation at 6,500 *g* for 10 min at 4°C, the harvested supernatant was aliquoted and stored at −80°C for subsequent experiments.

### Quantitative Reverse Transcriptase Polymerase Chain Reaction

An absolute quantification real-time PCR method for detecting the RdRp gene of CAstV group II was used as described previously (17) with minor modifications. In this study, we did not use a fluorogenic probe. We designed a specific primer pair of 5′-TGCAGATCCCGACGTAAAGG-3′ and 5′-CGGTCCATCCCTCTACCAGA-3′; the 133 bp fragment of the RdRp gene was amplified and cloned into pGEM-T vector (Promega, United States). After sequence confirmation, the standard plasmid was diluted from 1 × 10^8^ to 1 × 10^1^ copies/μl to generate the standard curve for the qRT-PCR assay. The specificity and sensitivity of the assay was evaluated as reported previously (17). The qRT-PCR was performed using PrimeScript RT Master Mix (TaKaRa, Dalian, China) following the manufacturer's instructions.

### Antiserum Generation

Virus preparation of NJ1701 (200 μl) which was free from bacteria and other viruses (16, 18) was inoculated into 6-week-old Balb/c mouse by peritoneum injection at an interval of 10 days. After the third immunization, serum was collected and identified as mouse-anti-CAstV antiserum.

### Confocal Microscopy

The protocol was the same as that of our previous report (19). Briefly, infected LMH cells on coverslips were fixed with 4% paraformaldehyde in PBS for 20 min at room temperature, permeabilized with 0.25% Triton X-100 for 5 min, washed with PBS, blocked with 2% BSA for 30 min, and incubated with mouse-anti-CAstV anti-serum (1:100) in PBS for 45 min at room temperature. The cells were washed in PBS, incubated with goat anti-mouse IgG conjugated with FITC (SIGMA, Shanghai, China) for 30 min at room temperature, and then stained with 10 μg/ml of Hoechst 33342 dye (SIGMA, Shanghai, China) at room temperature for an additional 10 min. The images were captured with a Leica SP2 confocal microscope.

### Genome Sequencing and Analysis

In order to sequence the genome of the NJ1701 isolate, total RNA was extracted from the virus-infected LMH cells using Total RNA Miniprep kit (AXYGEN, United States). The cDNA was synthesized by SuperScript™ III First-Strand Synthesis System (Invitrogen, United States) following the manufacturer's instructions. Overlapping PCR fragments were amplified across viral genome with primer sets designed against conserved regions of the CAstV sequences deposited in GenBank database (Table 1). All PCR products were cloned into pGEM-T vector (Promega, United States). At least two representative transformed clones of each PCR fragment were subjected to DNA sequencing by Gene Script Company (Nanjing, China).

The full-length genome sequence of the Chinese CAstV isolate (AAstV/Chicken/CHN/2017/NJ01) was deposited in GenBank with accession number MK746105. The sequence analysis was performed as described previously (18). The prediction of ORFs and the search for special sequences in the genome were conducted by using Lasergene7. The nucleotide and deduced amino acid sequences of ORFs obtained in this study were aligned using the Clustal W methods in MEGA 7.0 software, and phylogenetic trees were constructed using the neighbor-joining method with 1,000 bootstrap replicates in MEGA 7.0.

### Experimental Infection Study in Chicken Embryos

The experimental infection study was carried out as described previously with some modifications (16). In order to verify the proliferative capacity of the isolate in chicken embryos, 0.2 ml of the virus stock was inoculated into the chicken embryos via the yolk sac route and blindly passaged three times with allantoic fluid of the previous passage. Three chicken embryos were used each time. After that the qRT-PCR was carried out to detect the virus proliferation in each passage of the chicken embryos.

Moreover, to investigate the effect of the virus on the hatchability of chicken embryos, 60 7-day-old SPF chicken embryos (Merial, China) were divided into two groups. The infected group was inoculated with 0.2 ml of the virus stock via the yolk sac route. The negative control group was inoculated with 0.2 ml of cell culture medium. Embryonic viability was observed daily. The numbers of the hatched chicks were subjected to hatching rate calculation. The chicks hatched from infected group were euthanized and necropsied within 24 h after hatching. The virus distribution and viral copy numbers in different internal organs were examined by qRT-PCR. Animal infection experiments were approved by the Yangzhou University Animal Ethics Committee.

### Statistical Analyses

The results represent the means ± standard deviations (SD) of triplicate determinations. The significance of the variability between the trials was analyzed using GraphPad (version 5.0) software. Differences between samples were assessed by the Student's *t*-test, and *p* < 0.05 were statistically significant. The experiment was performed at least three times independently with similar results.

## Results

### Clinical Survey of Chicken Astrovirus in China

The RNA of 154 small intestine samples collected from chicken flocks in Guangdong, Jiangsu, Anhui, and Shandong Provinces were extracted for the 510 bp DNA amplification by RT-PCR method described in previous report (15). The agarose gel electrophoresis showed that DNA fragments about 510 bp were clearly visible (data not shown) in 127 of the 154 (82.5%) field samples tested. All of the PCR products were sequenced to confirm the presence of CAstV sequence. Subsequent phylogenetic analysis based on partial RdRp gene sequence revealed that five of seven sequences including NJ1701 belonged to Group II of *Avastrovirus* according to the classification criteria of previous publication (15).

### Efficient Isolation of the Chicken Astrovirus in LMH Cells

**Figure 1:**
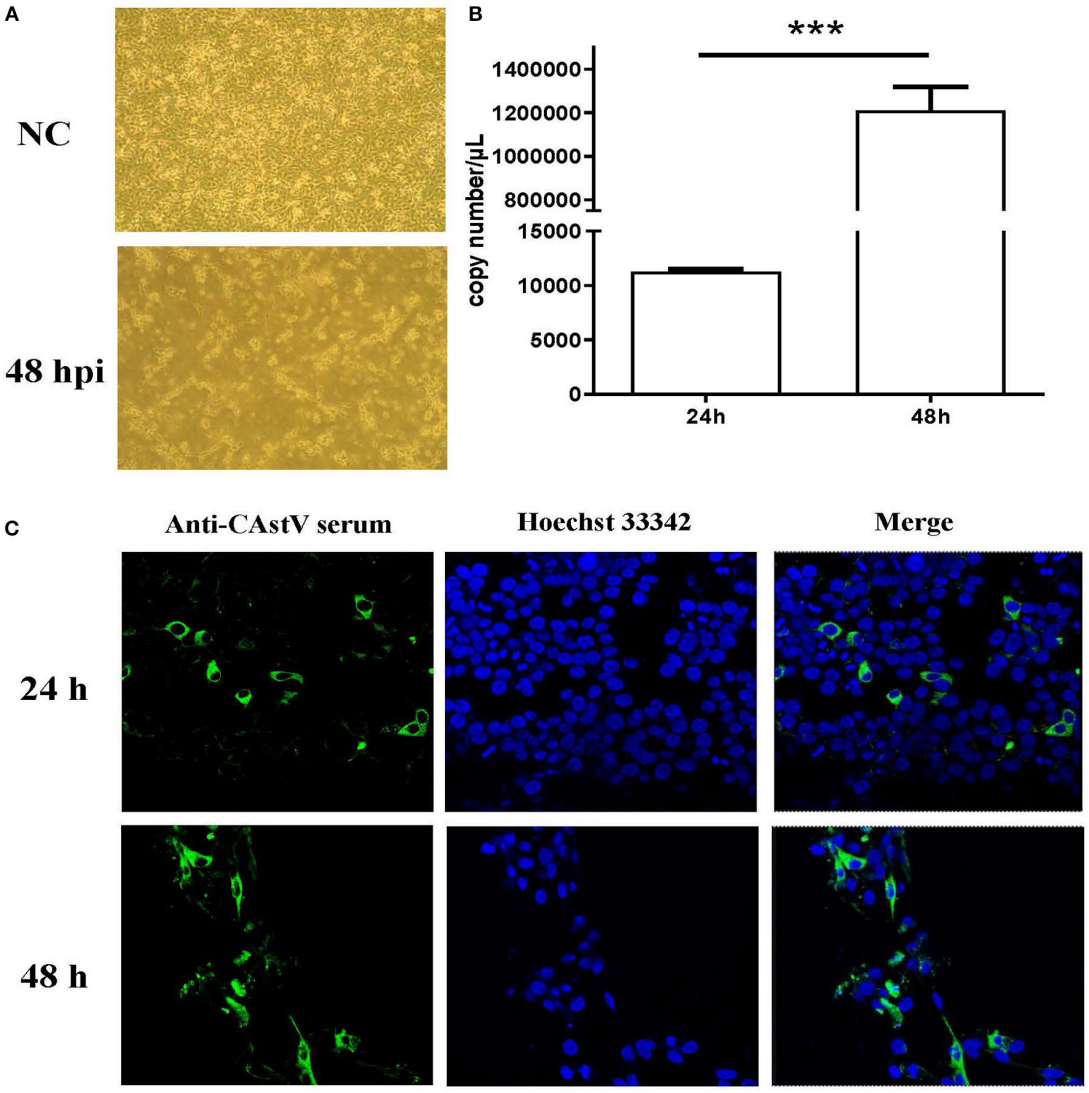
Isolation and characterization of CAstV NJ1701 isolate in LMH cell culture. After four passages, the virus was isolated and identified in LMH cells. **(A)** Uninfected LMH cells at 48 h post infection (p.i.) (NC, upper panel) and LMH cells infected with NJ1701 at 48 h p.i. (lower panel), which are detached with small and round cells present in the media (400×). **(B)** The viral gene copy numbers in cell supernatant were 1.13 × 10^4^ and 1.21 × 10^6^ copies/μl at 24 and 48 h p.i., respectively. Data were expressed as mean ± SD from three independent experiments. ***P < 0.001. **(C)** Indirect immunofluorescence assay was performed at 24 and 48 h post-infection with mouse-anti-CAstV serum. Immunofluorescence results of CAstV show cytoplasmic staining in the infected LMH cells.

The small intestines homogenate of 1-day-old chicks from a “Yellow” chicken flock with mild growth problem was used to isolate CAstV virus. After ruling out the other common enteric virus contamination by PCRs and RT-PCRs (16), the homogenate of the small intestine was inoculated onto LMH cells. The inoculated cells showed a cytopathic effect (CPE) beginning at fourth passage. At 48 h post infection (p.i.) detached small and round cells were predominant, representing almost 100% CPE (Figure 1A), which is similar to the CPE described by Kang et al. (20). qRT-PCR showed a significant increase in viral RNA from 24 to 48 h p.i. (Figure 1B). Confocal microscopy also showed an increase in viral antigen between 24 and 48 h p.i. (Figure 1C). We designated the first isolated Chinese chicken astrovirus as AAstV/Chicken/CHN/2017/NJ01, termed NJ1701 strain.

### Genome Sequence Analysis of Chicken Astrovirus Isolate

**Figure 2:**
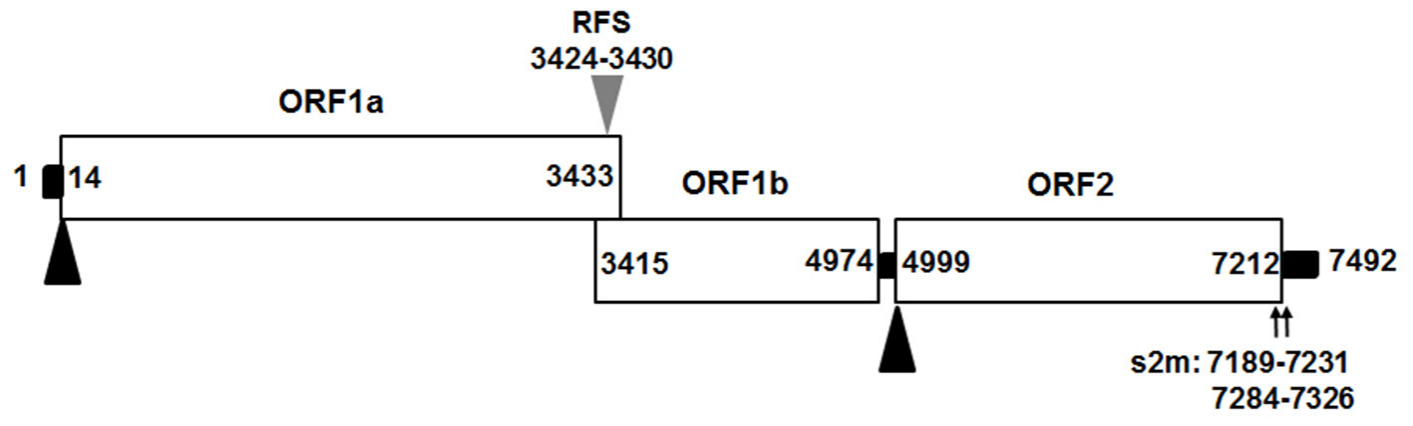
Predicted genome organization of CAstV NJ1701. Three predicated ORFs with their locations for CAstV NJ1701 are shown. The translation start sites of ORF1a and ORF2 are indicated by black triangles. The nucleotide position of the start site of the heptameric AAAAAAC (RFS) sequence are shown by gray triangles. Black bars are the untranslated regions and the 24-nt spacer between the stop and start codons of ORF1b and ORF2, respectively. ORF, open reading frame; RdRp, RNA-dependent RNA polymerase; RFS, ribosomal frameshift signal; s2m, stem-loop-II-motif.

We assembled the full-length genome of NJ1701 isolate following overlapping PCR and sequencing. The sequence of NJ1701 strain consisted of 7,492 nt, with the typical AstV genome structure of three ORFs: ORF1a, ORF1b, and ORF2 (Figure 2). Analysis of the nucleotide sequence similarity with other full-length genomes among chicken astroviruses revealed that NJ1701 has the closest similarity to recently published two Chinese CAstV strains CAstV /HBLP and CAstV/GDYHTJ (14) at the level of 98.7% and 97.5% (Table 2). The lowest identity is 69.4% with CAstV/Poland/G059. For the other avian astroviruses, the genome showed 44.3–51.0%, 50.5–60.9%, 27.5%, and 58.4% similarity with turkey astroviruses, duck astroviruses, avian nephritis viruses, and goose astroviruses, respectively. The amino acid sequences of ORF1a and ORF1b of NJ1701 shared the identities of 77.8–99.4% with the published sequences of other CAstVs (Table 2). Nevertheless, the amino acid sequence of ORF2 among these chicken astrovirus strains was very diverse, ranging between 37.8 and 99.9% similarities (Table 2).

**Table 2:** Comparisons of nucleotide and amino acid sequences of CAstV NJ1701 with selected representative astroviruses.

**Species**	**Virus**	**GenBank accession no**.	**Sequence identity (%)**				**Genetic distance**
			**Genome (nt)**	**ORF1a (aa)**	**ORF1b (aa)**	**ORF2 (aa)**	**ORF2 (aa)**
CAstV	CAstV/INDIA	KY038163	80.4	88.1	86.4	91.1	0.09
	CAstV /HBLP	MN725025.1	98.7	99.3	99.2	99.9	0.03
	CAstV/GDYHTJ	MN725026.1	97.5	98.4	99.4	99.7	0.04
	CAstV/GA2011	JF414802	77.6	88.0	87.2	84.2	0.16
	CAstV/CC	KX397575	78.5	88.1	87.2	88.9	0.12
	CAstV/CkP5	KX397576	78.6	88.1	87.2	89.1	0.12
	CAstV/Poland/G059	KT886453	69.4	87.6	86.4	38.0	0.62
	CAstV/4175	JF832365	76.0	87.6	77.8	71.2	0.20
	CAstV/FP3	JN582328	–	–	–	96.1	0.04
	CAstV/P22-18.800	AFK92941	–	–	–	37.8	0.60
	CAstV/VF08-29	AFK92938	–	–	–	84.2	0.16
	CAstV/612	AFK92940	–	–	–	37.9	0.61
	CAstV/VF08-48	AFK92949	–	–	–	38.3	0.60
	CAstV/VF08-56	AFK92942	–	–	–	37.9	0.60
TAstV	TAstV-1	Y15936	44.3	38.3	57.3	32.2	0.64
	TAstV-2	AF206663	51.0	48.0	68.2	37.7	0.63
DAstV	DAstV/C-NGB	FJ434664	60.9	51.9	71.8	36.4	0.63
	DAstV/SL1	KF753804	50.5	48.8	72.3	36.1	0.64
GAstV	GAstV/GD	MG934571	58.4	49.3	64.0	34.9	0.64
ANV	ANV-1/China	HM029238	27.5	30.2	53.1	25.8	0.75

### Phylogenetic Analysis of Isolated Chicken Astrovirus

**Figure 3:**
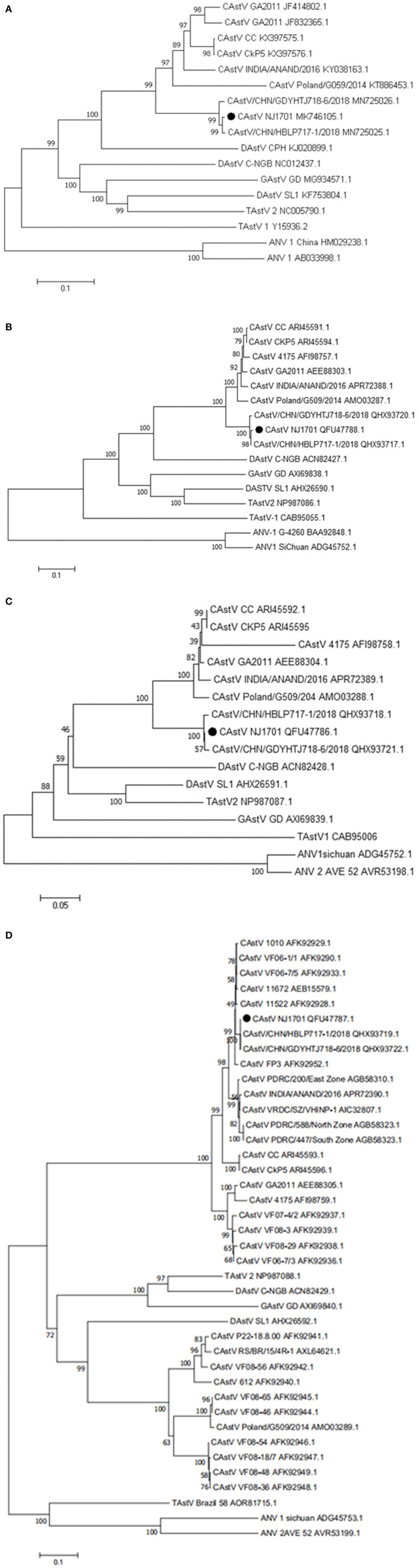
Phylogenetic relationships between CAstVs in this study and other avian astroviruses. The phylogenetic analysis of chicken astrovirus isolate NJ1701 was based on the nucleotide sequences of the complete genome **(A)** and on amino acid sequences of the complete ORF1a **(B)**, ORF1b **(C)**, and ORF2 **(D)** regions using neighbor-joining method with 1,000 bootstraps.

A phylogenetic analysis of the complete nucleotide sequence and all three ORFs was conducted to investigate the relationship of NJ1701 with other astroviruses. As shown in Table 2, NJ1701 had the highest percentage of identity with other two published Chinese strains, CAstV/HBLP and CAstV/GDYHTJ (14) for both nucleotide and amino acid sequences. All three Chinese strains formed a sister clade in the phylogenetic trees of complete genome sequence, ORF1a and ORF1b (Figures 3A–C). Further analysis in ORF2 phylogenetic tree revealed that in addition to the two strains of CAstV in China, NJ1701 is most closely related to the Chicken astrovirus isolate FP3, representing subgroup Bi (Figure 3D).

### Infection Experiments in Chicken Embryos

**Figure 4:**
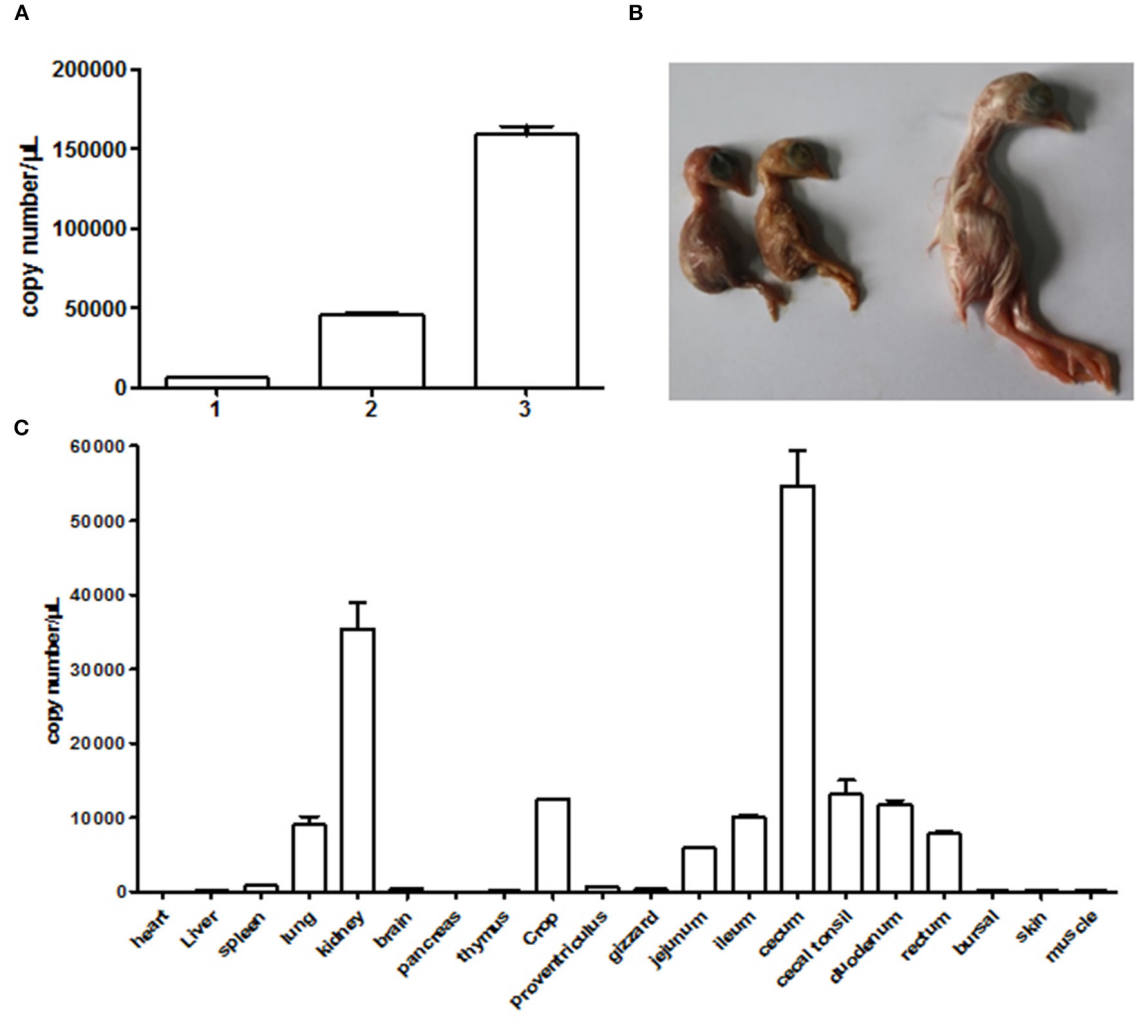
Infection experiments with CAstV NJ1701 in chicken embryos. **(A)** Viral gene copy numbers of allantoic fluid in embryos of different passages. The copy numbers of passages 1, 2, and 3 were 6.00 × 10^3^, 4.58 × 10^4^, and 1.59 × 10^5^, respectively. Data were expressed as mean ± SD from three independent experiments. **(B)** Ten days after infection at passage 3, 17-day-old infected embryos with growth depression (two of the left) were observed. The uninfected embryo (right) was used as control. **(C)** The viral copy numbers of different visceral organs from chicks at hatch in infected group were determined using qRT-PCR. Data were expressed as mean ± SD from three independent experiments.

We used the supernatant of cell culture at fourth passage to inject chicken embryos. The result in Figure 4A clearly showed that the viral gene copy number determined by qRT-PCR continued to increase in chicken embryos following blind passages. This revealed that the NJ1701 isolate replicated well in chicken embryos. Meanwhile, growth depression, dwarfism embryo caused by viral infection, was also observed (Figure 4B). In addition, the viral copy number of different organs from 1-day-old chicks in infected group indicated noteworthy information that the highest level of virus was in the cecum which has not been reported before. The kidney ranks the second, while the heart is the lowest one (Figure 4C). Sixty SPF chicken embryos were equally divided into two groups for investigating the effect of isolated virus on chick hatching rate. The level of hatching rate in infected group, 11/30 (36.67%), significantly decreased when compared with 100% in normal control group by analysis with Student's *t*-test. These results indicated that the NJ1701 isolate could replicate in chicken embryo and affect embryonic development, which suggested the possible link to the growth problems of chicken flocks in the field.

## Discussion

Astrovirus has high environmental stability and prevalence in over 80 host species (7). In recent years, increasing reports on diagnosis, pathogenesis, strain diversity, evolution, and emergence of astrovirus were published (1–3, 5, 9, 21). Except for the important clinical and agricultural significance, researchers concerned with the cross-species transmission of astrovirus, which eventually led to the evolution of the virus into zoonoses (3, 7). In fact, a few interspecies transmission and recombination events have been reported, which suggest that there is a risk for emergence of a novel recombinant astrovirus which could infect humans (3).

Until now, there are no medicines for treatment and vaccines to prevent transmission of astrovirus due to the lack of efficient cell culture systems, animal models, and full genome sequences for numerous astrovirus species (9). In the present study, one strain from “Yellow” chicken flock with mild growth problems was successfully isolated by culturing in LMH cell which provides a good tool for investigating the etiology, pathogenesis, and biology of CAstV and even developing inactivated vaccine in future studies. In chicken embryo experiment, both the detection of CAstV in allantoic fluids and reduction of hatchability combined with the positive results of qRT-PCR at hatch indicated the possibility of vertical transmission of CAstV NJ1701 isolate. At the same time, we also observed the dwarfism development of virus-inoculated chicken embryos as shown in Figure 4B. Further reduced hatching rate by virus inoculation indicates that the virus could affect the development of chicken embryos. Whether these results could explain the varying degrees of growth suppression in chicken flocks remains to be elucidated by detailed pathogenesis experiments. Virus distribution and copy numbers in different visceral organs of chicks at hatch demonstrated that the NJ1701 strain proliferates very well in the cecum, which is in disagreement with the usage of the small intestine of chicken for detection and isolation of CAstV (20). Virus detection results of clinical samples of 1-day-old chicks in our laboratory supported the finding that the cecum was indeed the organ with highest virus detection rate (data not shown). After the cecum, the kidney ranked the second in copy number of the viral gene. It is not surprising since the previous reports have mentioned that CAstV could infect organs outside of the enteric tract including the kidneys (9).

We assembled a full-length genome of the viral isolate AAstV/Chicken/CHN/2017/NJ01 using overlapping PCR with our own designed primers. This genome sequence will help scientific researchers to investigate the etiology, epidemiology, evolution, and pathogenesis of CAstV in China. The sequence of the AAstV/Chicken/CHN/2017/NJ01 strain consisted of 7,492 nt. It shares a similar genetic organization with other astroviruses (Figure 2). The phylogenetic tree analysis of the complete nucleotide sequence revealed that NJ01 strain belongs to the Chinese CAstV branch. It only has 69.4–80.4% identity with seven other published full genome sequence of CAstVs in GenBank. A similar result was observed in ORF1a and ORF1b gene analysis. Previous reports have suggested that the ORF2 (capsid gene) is the most hypervariable region associated with antigenicity (21, 22), and it is normally diverse in sequences between CAstV isolates in different countries (6, 8, 23). Interestingly, the ORF2 amino acid sequence identity between NJ1701 and UK isolate FP3 is very high, reaching 96.1%. The other two Chinese strains have very high homology with FP3 also. Due to the lack of more gene sequence information of CAstV/FP3, we cannot further analyze and determine the relationship between the Chinese strains and the FP3 strain in UK. Whether the Chinese strains and the FP3 strain originated from the same ancestor needs the full-length genome of CAstV/FP3 for clarification.

In summary, this is the first demonstration of successful isolation of chicken astrovirus AAstV/Chicken/CHN/2017/NJ01 in “Yellow” chicken flock in China by culturing in LMH cells. We have shown that the virus has the typical genomic characteristics of avian astroviruses. Based on the genetic analysis of ORF2 encoded capsid region, the isolate should be assigned as a member within CAstV subgroup Bi. The result of RT-PCR on samples collected from four different provinces revealed the wide spread of chicken astrovirus in China. Moreover, it is noteworthy that the highest level of viral gene copy numbers present in different organs of chick at hatch is in the cecum, which has not been reported before. In future studies, sequencing more isolates and *in vivo* experiments are required to study the origin, variation, pathogenesis, and potential zoonotic infection of the novel chicken astrovirus in China.

## Data Availability Statement

The datasets generated for this study can be found online at the GenBank repository, with code MK746105.

## Ethics Statement

The animal study was reviewed and approved by the Animal Care and Use Committee of Yangzhou University.

## Author Contributions

KQ and AQ designed the study. WZ and ZW carried out the experiments, analyzed the data, and drafted the manuscript. KQ supervised all the experiments and participated in the data analysis. YY discussed and revised the final manuscript. All authors contributed to the article and approved the submitted version.

## Conflict of Interest

The authors declare that the research was conducted in the absence of any commercial or financial relationships that could be construed as a potential conflict of interest.

## References

[1] Cortez V Meliopoulos VA Karlsson EA Hargest V Johnson C Schultz-Cherry S. Astrovirus biology and pathogenesis. Ann Rev Virol. (2017) 4:327–48. 10.1146/annurev-virology-101416-041742 28715976

[2] Johnson C Hargest V Cortez V Meliopoulos VA Schultz-Cherry S. Astrovirus pathogenesis. Viruses. (2017) 9:22. 10.3390/v9010022 28117758 PMC5294991

[3] Wohlgemuth N Honce R Schultz-Cherry S. Astrovirus evolution and emergence. Infect Genet Evol. (2019) 69:30–7. 10.1016/j.meegid.2019.01.009 30639546 PMC7106029

[4] Appleton H Buckley M Robertson MH Thom BT. A search for faecal viruses in new-born and other infants. J Hyg. (1978) 81:279–83. 10.1017/S0022172400025110 212477 PMC2129777

[5] Perot P Lecuit M Eloit M. Astrovirus diagnostics. Viruses. (2017) 9:10. 10.3390/v9010010 28085120 PMC5294979

[6] Sajewicz-Krukowska J Domanska-Blicharz K. Nearly full-length genome sequence of a novel astrovirus isolated from chickens with 'white chicks' condition. Arch Virol. (2016) 161:2581–7. 10.1007/s00705-016-2940-6 27339687 PMC4987400

[7] Donato C Vijaykrishna D. The broad host range and genetic diversity of mammalian and avian astroviruses. Viruses. (2017) 9:102. 10.3390/v9050102 28489047 PMC5454415

[8] Pantin-Jackwood MJ Strother KO Mundt E Zsak L Day JM Spackman E. Molecular characterization of avian astroviruses. Arch Virol. (2011) 156:235–44. 10.1007/s00705-010-0849-z 21069394

[9] Smyth VJ. A review of the strain diversity and pathogenesis of chicken astrovirus. Viruses. (2017) 9:29. 10.3390/v9020029 28208602 PMC5332948

[10] Bulbule NR Mandakhalikar KD Kapgate SS Deshmukh VV Schat KA Chawak MM. Role of chicken astrovirus as a causative agent of gout in commercial broilers in India. Avian Pathol. (2013) 42:464–73. 10.1080/03079457.2013.828194 24015918

[11] Long KE Ouckama RM Weisz A Brash ML Ojkic D. White chick syndrome associated with chicken astrovirus in Ontario, Canada. Avian Dis. (2018) 62:247–58. 10.1637/11802-012018-Case.1 29944402

[12] Sun N Yang Y Wang GS Shao XQ Zhang SQ Wang FX. Detection and characterization of avastrovirus associated with diarrhea isolated from minks in China. Food Environ Virol. (2014) 6:169–74. 10.1007/s12560-014-9155-3 24915926

[13] Xue J Han T Xu M Zhao J Zhang G. The first serological investigation of chicken astrovirus infection in China. Biologicals. (2017) 47:22–4. 10.1016/j.biologicals.2017.03.005 28347631

[14] Xue J Han T Zhao Y Yang H Zhang G. Complete genome sequence and phylogenetic analysis of novel avastroviruses circulating in China from 2016 to 2018. Virus Res. (2020) 278:197858. 10.1016/j.virusres.2020.197858 31904408

[15] Smyth VJ Jewhurst HL Adair BM Todd D. Detection of chicken astrovirus by reverse transcriptase-polymerase chain reaction. Avian Pathol. (2009) 38:293–9. 10.1080/03079450903055397 19937514

[16] Nunez LF Parra SH Mettifogo E Catroxo MH Astolfi-Ferreira CS Piantino Ferreira AJ. Isolation of chicken astrovirus from specific pathogen-free chicken embryonated eggs. Poult Sci. (2015) 94:947–54. 10.3382/ps/pev086 25805833

[17] Le Cann P Ranarijaona S Monpoeho S Le Guyader F Ferre V. Quantification of human astroviruses in sewage using real-time RT-PCR. Res Microbiol. (2004) 155:11–5. 10.1016/j.resmic.2003.09.013 14759703

[18] Zhang X Ren D Li T Zhou H Liu X Wang X. An emerging novel goose astrovirus associated with gosling gout disease, China. Emerg Microbes Infect. (2018) 7:152. 10.1038/s41426-018-0153-7 30185786 PMC6125322

[19] Qian K Gao AJ Zhu MY Shao HX Jin WJ Ye JQ. Genistein inhibits the replication of avian leucosis virus subgroup J in DF-1 cells. Virus Res. (2014) 192:114–20. 10.1016/j.virusres.2014.08.016 25197039

[20] Kang KI Linnemann E Icard AH Durairaj V Mundt E Sellers HS. Chicken astrovirus as an aetiological agent of runting-stunting syndrome in broiler chickens. J Gen Virol. (2018) 99:512–24. 10.1099/jgv.0.001025 29458661

[21] Arias CF DuBois RM. The astrovirus capsid: a review. Viruses. (2017) 9:15. 10.3390/v9010015 28106836 PMC5294984

[22] Smyth VJ Todd D Trudgett J Lee A Welsh MD. Capsid protein sequence diversity of chicken astrovirus. Avian Pathol. (2012) 41:151–9. 10.1080/03079457.2011.652938 22515534

[23] Patel AK Pandit RJ Thakkar JR Hinsu AT Pandey VC Pal JK. Complete genome sequence analysis of chicken astrovirus isolate from India. Vet Res Commun. (2017) 41:67–75. 10.1007/s11259-016-9673-6 28012117 PMC7088555

